# Beef-Derived Peptides Mediated Desensitization of Bitter Taste Receptor T2R14 Through GPCR Kinase 2

**DOI:** 10.3390/nu18060901

**Published:** 2026-03-12

**Authors:** Nisha Singh, Julia Drube, Carsten Hoffmann, Rotimi Emmanuel Aluko, Prashen Chelikani

**Affiliations:** 1 Department of Oral Biology, Rady Faculty of Health Sciences, University of Manitoba, Winnipeg, MB R3E 0W2, Canada; 2 Manitoba Chemosensory Biology Research Group, University of Manitoba, Winnipeg, MB R3E 0W2, Canada; rotimi.aluko@umanitoba.ca; 3 Institut für Molekulare Zellbiologie, CMB—Center for Molecular Biomedicine, Universitätsklinikum Jena, Friedrich-Schiller-Universität Jena, Hans-Knöll-Straße 2, D-07745 Jena, Germany; 4 Department of Food and Human Nutritional Sciences, University of Manitoba, Winnipeg, MB R3T 2N2, Canada; 5 Richardson Center for Food Technology and Research, University of Manitoba, Winnipeg, MB R3T 2N2, Canada; 6 Department of Biochemistry and Medical Genetics, Rady Faculty of Health Sciences, University of Manitoba, Winnipeg, MB R3E 0W2, Canada; 7 Department of Physiology and Pathophysiology, Rady Faculty of Health Sciences, University of Manitoba, Winnipeg, MB R3E 0W2, Canada

**Keywords:** food peptides, bitter taste receptor, cAMP, calcium mobilization, G protein-coupled receptor kinases, desensitization, inhibition

## Abstract

**Background/Objectives:** Humans have at least 26 bitter taste receptors (T2Rs), and among these, bitter taste receptor 14 (T2R14) is highly expressed in both oral and extraoral tissues. Over 100 bitter ligands can activate T2R14, including hormones, vitamins, plant compounds, and peptides. Previous studies suggest that bitter tastants such as quinine and caffeine can inhibit G protein-coupled receptor kinases (GRKs) and delay T2R signal termination. Our earlier research showed that peptides from alcalase and chymotrypsin hydrolysates of beef proteins inhibited quinine-dependent calcium release through T2R4, with AGDDAPRAVF and ETSARHL showing the greatest effectiveness. However, the effect of these antagonistic peptides on other T2Rs, such as T2R14 signaling, remains unknown. This study aimed to evaluate the ability of these beef protein-derived peptides to activate or inhibit T2R14 signaling and the involvement of GRK2 in signal termination. **Methods and Results:** Our results indicate that the above two antagonist peptides significantly inhibit T2R14 activity. Furthermore, GRK2 knockout in HEK cells stably expressing T2R14 decreases intracellular calcium release, as measured by the area under the curve (AUC), and also delays the fall time (indication of desensitization) of the calcium response when exposed to the T2R14 agonist diphenhydramine (DPH) or beef protein-derived agonist peptide TMTL. Next, we measured the effects of these ligands on cAMP accumulation, and our results suggest no significant change in cAMP levels upon treatment with beef protein-derived peptides. **Conclusions:** Thus, this study showed that beef protein-derived peptides can function as both T2R inhibitors and mediate T2R14 desensitization through GRK2 signaling. These antagonistic food protein-derived peptides inform strategies to enhance nutrition, such as promoting healthier food choices by reducing bitterness and thereby improving the palatability of health-promoting bitter foods, such as fruit and vegetable extracts, as well as bitter medications.

## 1. Introduction

Taste perception is a vital sensory process that enables organisms to assess the quality of food, with food-derived peptides playing a crucial role as modulators of this process. Food-derived peptides can trigger taste sensations—such as bitterness and umami—by interacting with specific receptors, notably bitter taste receptors (T2Rs) and umami receptors (T1R1/T1R3) [1,2]. Food-derived antagonistic peptides for T2Rs can be used to reduce unpleasantness and thereby enhance the palatability of health-promoting bitter foods, such as fruit and vegetable extracts, as well as bitter compounds in medications.

Taste receptors, which are predominantly G protein-coupled receptors (GPCRs), mediate the detection of taste signals such as sweet, umami, and bitter by responding to various ligands, including food-derived peptides. In humans, more than 30 GPCRs are known to mediate taste perception, with 26 of these receptors and their pseudogenes (T2Rs or TAS2Rs) involved in sensing bitter taste [3,4,5,6], and 3 receptors (T1Rs) sensing sweet and umami tastes [7,8]. Desensitization of GPCRs is a crucial physiological feedback mechanism that protects against both acute and chronic receptor over-stimulation. Research indicates that prolonged exposure to certain tastants can lead to taste desensitization through mechanisms such as receptor downregulation, protein degradation via the ubiquitin-proteasome pathway, and alterations in synaptic connections, ultimately influencing food preferences [9,10].

Sustained or repetitive agonist exposure causes loss or desensitization of GPCR signaling. The earliest event in GPCR desensitization is receptor phosphorylation [11]. GPCR phosphorylation is mediated by two families of protein kinases: GPCR kinases (GRK) and second messenger-dependent protein kinases such as protein kinase C (PKC) and protein kinase A (PKA). GRKs phosphorylate only the agonist-activated form of GPCRs and promote arrestin binding, which uncouples the receptor from the G-protein, resulting in GPCR signal termination [12]. Multiple studies showed that PKA does not phosphorylate T2Rs, leaving PKC and GRKs as potential candidates for T2R phosphorylation [13,14]. The mechanisms by which various T2R agonists, antagonists and GRK/β-arrestin complexes interact during signaling and receptor desensitization are not fully understood [15,16]. Among these, T2R14 stands out due to its high expression in oral tissues and many other extraoral sites [17,18,19], where it plays a role in innate immunity by responding to specific bacterial and fungal compounds [20,21].

Similar to other GPCRs, endogenous T2Rs in human airway smooth muscle (ASM) cells undergo desensitization after exposure to compounds like quinine and saccharin [14]. It has been shown that GRK2 phosphorylates Ser/Thr residues at the receptor’s C-terminus or intracellular loop 3 (ICL3), which mediates short-term desensitization of T2R14 when stimulated by diphenhydramine (DPH) [22]. Studies have also revealed that residues in ICL3 and the C-terminus contribute differently to receptor desensitization, β-arrestin recruitment, and internalization. Food peptides can induce signaling bias in taste GPCRs by selectively activating specific G proteins or downstream signaling elements. For example, T2Rs can be differentially activated by peptides, leading to unique signaling responses that are modulated by G proteins, such as gustducin or Gi, and influenced by GRKs. GRKs are critical for desensitizing T2Rs by phosphorylating them upon activation, encouraging β-arrestin binding and receptor internalization [22]. This process ultimately regulates taste sensitivity and its desensitization.

Previous research has shown that certain amphipathic tastant molecules, including some peptides with both hydrophobic and hydrophilic parts, can influence GRK-mediated desensitization [13]. Specifically, amphipathic tastants may inhibit GRK2 activity in taste cells, delaying receptor desensitization and potentially extending taste signaling [23]. Earlier studies suggested that GRK2, 3, 5, and 6, along with taste receptors such as T2R4 and T1R3, are present in taste buds [13]. Currently, there is no clear evidence that food-derived peptides inhibit multiple T2Rs, their effect on GRK-mediated T2R signaling or alter taste signaling outcomes via cAMP or calcium. This study aims to investigate whether beef protein-derived peptides can inhibit T2R14 signaling and modulate T2R14 signal termination through GRK2.

## 2. Materials and Methods

### 2.1. Materials

GenScript Inc. (Piscataway, NJ, USA) carried out the de novo synthesis of peptides (>96% purity). 6-methoxy flavanone (6-MF) and diphenhydramine (DPH) were purchased from Alfa Aesar and Sigma-Aldrich (St. Louis, MO, USA), respectively. Calcium-sensitive dye Fluo-4 NW kit (F36206) was purchased from Life Technologies (Carlsbad, CA, USA). The Gαi cAMP kit was purchased from Revvity, Mississauga, ON, Canada. Cell culture media (DMEM/F12), supplements, dissociation reagent (Trypsin), and phosphate-buffered saline (PBS) were purchased from Invitrogen (Burlington, ON, Canada).

### 2.2. Molecular Biology and Cell Culture

The Human Embryonic Kidney (HEK293T) cell line was obtained from ATCC (Manassas, VA, USA). A human *TAS2R14* gene, codon-optimized for expression in mammalian cells and tagged at the N-terminus with a FLAG epitope, was cloned into the KpnI-NotI site of the pcDNA3.1/Hygro (+) expression vector. This construct was commercially synthesized (GenScript Inc., Piscataway, NJ, USA) as previously described in Reference [24]. Stable HEK293T cell lines expressing T2R14 were generated using selection medium containing 200 μg/mL hygromycin, following the procedure outlined in previous publications [25]. The CRISPR/Cas9 HEK293 cells (Control), ΔGRK2 HEK293 cells and GRK2 plasmid used in the study were obtained from Dr. Carsten Hoffman’s lab under a material transfer agreement (MTA). The knockout (KO) was confirmed by Western blot analysis [26].

### 2.3. Flow Cytometry Analysis

Surface expression of FLAG-tagged T2R14 WT receptor and ΔGRK2 HEK293 + T2R14 cells was measured with a BD FACS Canto flow cytometer as described before [27]. In brief, 50,000 cells were washed with ice-cold FACS buffer (0.5% BSA in PBS) and incubated for 1 h on ice with APC-conjugated mouse monoclonal anti-FLAG M2 antibody (dilution 1:500). Afterward, the cells were washed three times with FACS buffer and resuspended in 300 μL of buffer. Fluorescence intensity was assessed using the FACS Canto analyzer (BD Biosciences, San Jose, CA, USA), and mean fluorescence intensity values were recorded. Results are shown as a percentage of T2R14 cell surface expression relative to un-transfected (HEK 293 and Δ GRK2 HEK 293) cells.

### 2.4. Determination of Intracellular Calcium Mobilization

The potential of identified food peptides to activate or inhibit bitter taste receptors was evaluated by measuring intracellular calcium mobilization using a Fluo-4 NW calcium assay (Invitrogen, Burlington, ON, Canada), as previously described [24,28]. HEK293T cells stably expressing T2R14 were plated at 1 × 10^5^ cells per well in 96-well BD Falcon Biolux plates and incubated at 37 °C with CO_2_ for 16–18 h. After incubation, the culture medium was replaced with Fluo-4 NW dye solution (lyophilized dye reconstituted in 10 mL assay buffer, 1× Hanks’ balanced salt solution, 20 mM HEPES, plus 100 μL of 2.5 mM probenecid to minimize dye leakage) and incubated for 40 min at 37 °C, then 30 min at room temperature. Ca^2+^ mobilization was quantified as relative fluorescence units (RFUs) using a FlexStation-3 microplate reader (Molecular Devices, San Jose, CA, USA) (excitation: 494 nm; emission: 516 nm). Receptor activation was assessed through Ca^2+^ signaling in response to individual food-derived peptides (1 mM). DPH (0.5 mM) was used as an agonist for T2R14, and 6-MF (120 μM) was used as an antagonist for T2R14. The agonist was used alone or in combination with peptides to evaluate their modulatory effects in 50 µL volumes automatically added by the Flex station 3 pipettor head. Basal Ca^2+^ was monitored for 20 s, then ligands were added after 20 s, and readings were taken for another 300 s every 4 s intervals. Absolute RFUs were calculated as the difference between peak (Max) and baseline (Min) values after stimulation. ΔRFUs were determined by subtracting negative control signals from experimental group signals. Results were obtained from three independent experiments, each performed in triplicate, and analyzed using GraphPad Prism v9.01 (GraphPad Software, San Diego, CA, USA). To assess the kinetic characteristics of the calcium response, we measured the rise and decay times [29], defined as the intracellular calcium response required to reach peak and return to baseline following activation by bitter peptides or agonists.

### 2.5. Second Messenger cAMP Assays

Activation of the Gi pathway results in the inhibition of cAMP, which was measured using the HTRF-based CisBio/Revvity cAMP Gαi detection kit (Mississauga, ON, Canada) [20]. HEK293, WT and Δ GRK2 cells stably expressing T2R14, 10,000 cells per well, were plated in a low-volume 96-well plate using assay 1× stimulation buffer in 5 μL volume. After a 15 min pretreatment with 1 mM food peptides at 37 °C, the cells were stimulated with 500 μM DPH in assay buffer for 15 min. Subsequently, cells were stimulated with 1 μM forskolin. Later, cells were incubated with cAMP Eu-cryptate and anti-cAMP-d2 antibody for 1 h at room temp. Standards were prepared according to the manufacturer’s instructions. After one hour, the HTRF ratio was measured, and cAMP concentrations (nM) were calculated by interpolating the data with a standard curve in GraphPad Prism v10.6.

### 2.6. Statistical Analysis

All experimental results are presented as the average values ± Standard error of the mean (SEM) from at least three independent experiments performed in triplicate. ΔRFU was plotted as a function of agonist concentration and analyzed by non-linear regression (four parameters fit). IC_50_ values were obtained from fitting parameters in GraphPad Prism. For analyzing the kinetics of calcium signaling ‘Baseline then rise-and-fall to baseline time course’ was used in Graph Pad Prism 10.6. * Indicates differences are significant at *p* < 0.05, ** indicates differences are significant at *p* < 0.01, *** indicates differences are significant at *p* < 0.001.

One-way analysis of variance (ANOVA) was applied when comparing three or more groups.

## 3. Results

### 3.1. Screening of Beef Peptides Against T2R14 Activity

**Figure 1:**
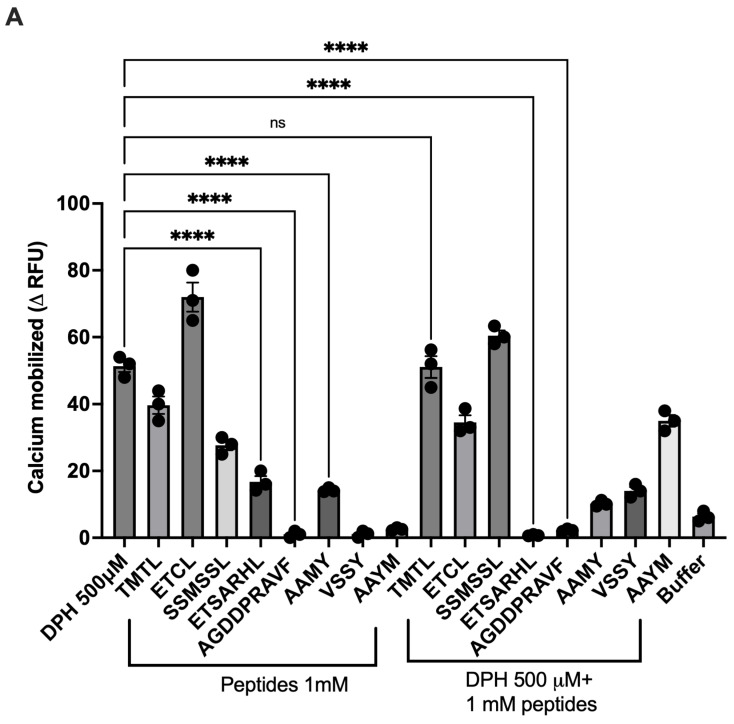

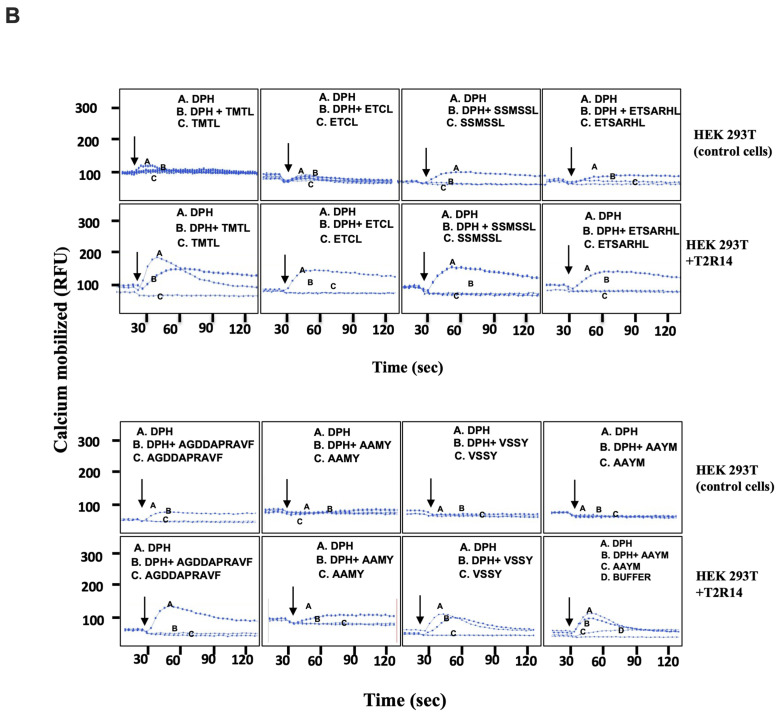
T2R14 activation and inhibition by beef-derived peptides. (**A**). Calcium mobilization in HEK293T cell stably expressing T2R14 and treated with DPH and/or beef peptides. The T2R14 expressing cells were treated with synthesized peptides (1 mM) and DPH (0.5 mM) alone or in combination. The calcium responses of cells treated with buffer are used as the control. Statistically significant values are shown by an asterisk, ^ns^ *p* > 0.99 and **** *p* < 0.0001. Bar graph data are ±SEM (n = 3) experiments performed in triplicate. For statistical comparison, HEK 293 T cells expressing T2R14 treated with peptides alone and with DPH and peptides were compared to DPH alone treatments. (**B**). Representative raw calcium traces for peptides, DPH and DPH+ peptides in HEK 293 T cells (control cells) and HEK 293 T cells stably expressing T2R14 (HEK 293 T+ T2R14). Arrows indicate the time point (20 s) at which the ligands were added.

T2R14 is a well-studied taste receptor that is significantly expressed in oral and extraoral tissues [18,30,31,32], and is known to be activated by diverse groups of ligands, including hormones, bacterial and fungal compounds, flavanones, and terpenoids [20,33]. Our previous study suggests that a number of beef-derived peptides, including ETSARHL, ETCL, AGDDAPRAVF, and AAMY, reduced quinine-induced calcium mobilization (ΔRFU) in HEK293 cells expressing T2R4 [34]. Therefore, in the current study, we examined the effect of these agonistic and antagonistic peptides on T2R14 activity. DPH activates T2R14 in HEK293 cells with an EC_50_ of 566 ± 72 µM [28]; hence, this DPH concentration (0.5 mM) was selected for T2R14 activation and further screening of beef peptide activity. Among all of the beef peptides analyzed for T2R14 activity, 1 mM of peptides ETSARHL and AGDDAPRAVF significantly decreased calcium mobilization when co-incubated with 0.5 mM DPH (Figure 1A). Representative raw calcium traces for peptide (TMTL, ETCL, SSMSSL, ETSARHL, AGDDAPRAVF, AAMY, VSSY, and AAYM)-treated T2R14, DPH-treated T2R14, and DPH co-incubated with peptide responses are shown in Figure 1B.

### 3.2. Antagonist Efficacy of Beef Peptides on DPH-Activated T2R14

**Figure 2:**
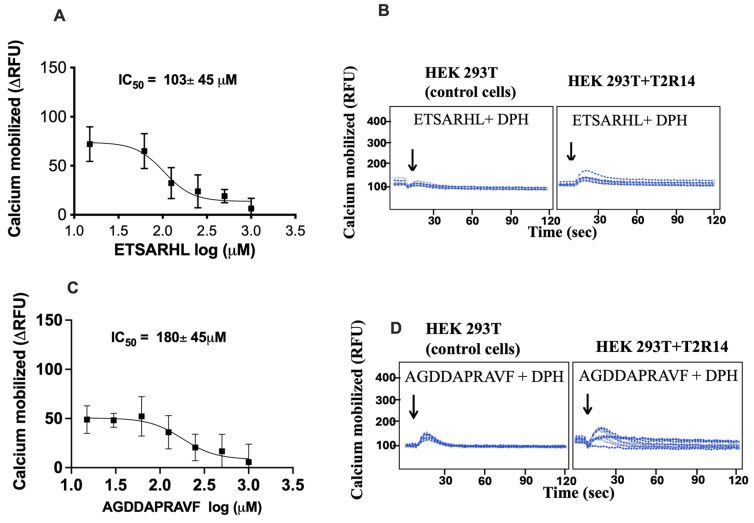
Competition assays for T2R14 and beef peptides. (**A**–**D**). Concentration-dependent responses of T2R14-expressing cells stimulated with 0.03–1 mM of ETSARHL or AGDDAPRAVAVF in the presence of DPH (0.5 mM). Calcium mobilization decreased with increasing concentrations of ETSARHL and AGDDAPRAVF, with IC_50_ values of 103 ± 45 µM and 180 ± 45 µM, respectively. Representative calcium traces of T2R14 expressed in HEK293T cells stimulated with DPH, DPH + ETSARHL, and DPH + AGDDAPRAVF. Arrows indicate the time point (20 s) at which the ligands were added.

Competition calcium mobilization assays on T2R14 were performed using two potent antagonistic peptides, ETSARHL and AGDDAPRAVF, to determine their inhibitory concentrations (IC_50_). The results indicated that these peptides inhibited the DPH (0.5 mM) response in a concentration-dependent manner, with ETSARHL and AGDDAPRAVF exhibiting IC_50_ values of 103 μM and 180 μM, respectively (Figure 2A,C). Raw calcium traces for peptides, ETSARHL and AGDDAPRAVF, on DPH-mediated T2R14 responses are presented in Figure 2B,D.

### 3.3. Effect of GRK2 Deletions on T2R14 Signaling Kinetics Following Treatment with Beef Peptides

There are seven isoforms of GRKs (1–7), with four of them, GRKs 2, 3, 5, and 6, being ubiquitously expressed [35,36,37]. Previous studies suggested that GRKs are involved in the desensitization process caused by many sweet and bitter tastants [38]. Earlier research also reported that GRK2 is involved in the phosphorylation and desensitization of T2R14 [22]. Therefore, in the present study, calcium signaling kinetics were examined upon co-treatment of beef peptides with DPH in the presence and absence of GRK2. First, cell surface expression was assessed by flow cytometry in both cell types (T2R14 and GRK2 + T2R14) and compared with their respective control cells (Figure S1). Next, to assess the involvement of GRK2, the pharmacological inhibitor cmpd 101 [25] was used, and the results are shown in Figure S2.

**Figure 3:**
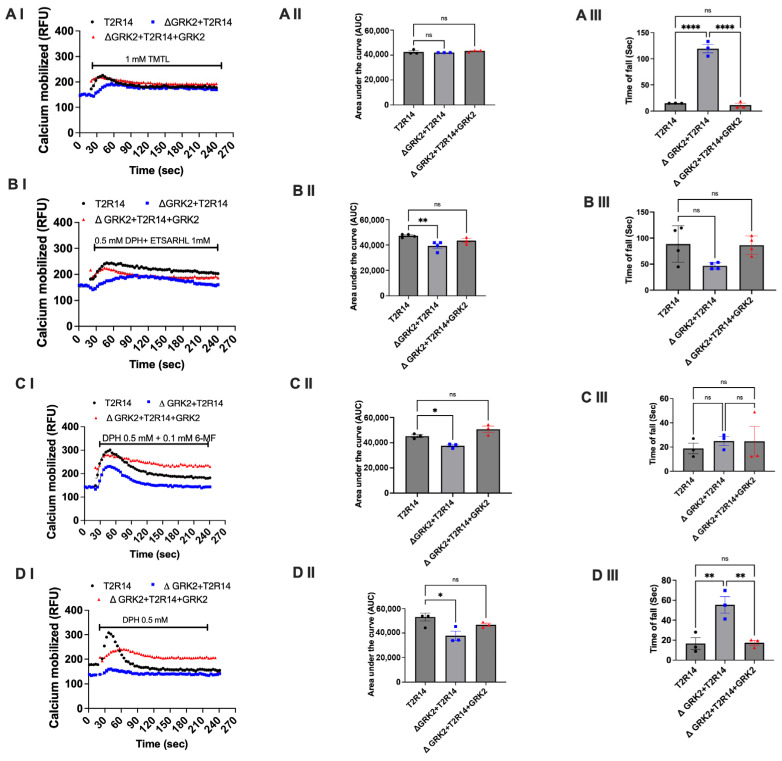
Effect of GRK2 on T2R14 signal kinetics with agonistic and antagonistic beef peptides. HEK293 and ΔGRK2 cells stably expressing T2R14 were treated with DPH (0.5 mM) and beef peptides (1 mM alone or in combination). Next, to assess the GRK2 rescue effect, HEK293 ΔGRK2 cells stably expressing T2R14 were transiently transfected with a GRK2 plasmid using Lipofectamine 2000. After 24 h of transfection, cells (1 × 10^5^/96 well) were treated with DPH (0.5 mM) and beef peptides (1 mM alone or in combination). Calcium mobilization was assayed using the Fluo-4NW dye and continuously monitored over 300 s using a Flex Station 3 multimode plate reader. The results are presented as relative fluorescence units (RFU) (**AI**–**DI**). (**AII**–**DII**), area under the curve (AUC). The data represent the SEM of at least three independent experiments performed in triplicate. (**AIII**–**DIII**), Time of fall (indication of desensitization) was plotted using the kinetics of calcium signaling. ‘Baseline then rise-and-fall to baseline time course’ was used in Graph Pad Prism 10.6. ^ns^ *p* > 0.99, * *p* < 0.05, ** *p* < 0.01 and **** *p* < 0.0001.

To demonstrate GRK2 specificity, we used CRISPR/Cas9-deleted GRK2 (ΔGRK2) HEK cells stably expressing T2R14. Two beef peptides, an agonistic peptide (TMTL) and an antagonistic peptide (ETSARHL), were selected. The results indicated that 1 mM treatment with the agonistic peptide TMTL in ΔGRK2-expressing T2R14 cells showed no significant change in calcium response, as shown by the area under the curve (AUC) (Figure 3A(I,II)). However, 1 mM TMTL treatment in ΔGRK2-expressing T2R14 cells required more time to fall, as shown in the calcium response (Figure 3A(III)). The antagonistic beef peptide ETSARHL led to less calcium mobilization induced by DPH, as shown by the curve and AUC (Figure 3B(I,II)). This antagonistic beef peptide ETSARHL did not alter the signaling kinetics of T2R14, whether or not GRK2 was present (Figure 3B(III)). Similarly, the synthetic T2R14 antagonist 6-MF also did not cause any change in T2R14 signaling (Figure 3C(I–III)) except for the calcium response shown by the area under the curve (AUC). However, 0.5 mM DPH treatment in ΔGRK2-expressing T2R14 cells required more time to fall, as shown in the calcium response (Figure 3D(III)). Next, a GRK2 rescue experiment was performed to test whether reconstitution restores the wild-type Ca^2+^ response kinetics. The results suggested that expressing GRK2 in ΔGRK2-T2R14 cells restores the wild-type Ca^2+^ response kinetics (Figure 3A–D). Further, we tested whether food-derived peptides can alter intracellular calcium kinetics directly acting through GRK2 by using native HEK293 and ΔGRK2 cells; the results were inconclusive (Figure S3).

### 3.4. Effect of Preincubation with Beef Peptides (1 mM) on cAMP Formation in the Absence of GRK2

Besides the effect on intracellular calcium levels, the interaction of T2Rs with ligands might cause changes in cAMP concentrations. Recently, the involvement of cAMP has been shown for signaling by bitter-tasting caffeine, where treating the human gastric cancer cells (HGT-1) with 3 mM caffeine led to proton secretion via TAS2R43 and an increase in intracellular cAMP levels [39]. Co-stimulation of the cells with the adenylyl cyclase inhibitor NKY80 [40] decreased cAMP production and reduced caffeine’s effect on proton secretion [39]. Likewise, sweeteners that affect serotonin release and proton secretion in HGT-1 cells via the taste receptor TAS1R3 increased intracellular cAMP levels, whereas simultaneous treatment with NKY80 attenuated both effects [41].

**Figure 4:**
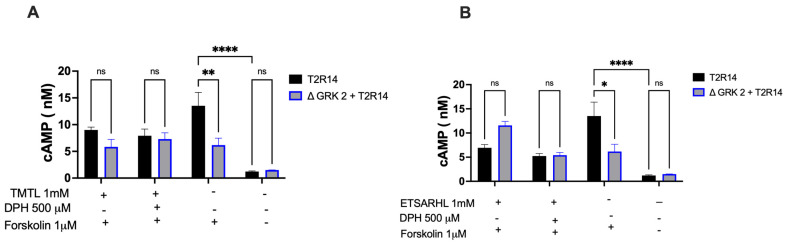
Effect of beef peptides preincubation on cAMP formation with or without GRK2. (**A**,**B**). HEK293 and ΔGRK2 cells stably expressing T2R14 (10,000 cells) were preincubated with beef peptides for 10 min. Cells were stimulated with 500 μM DPH for 15 min, and cAMP levels were measured by HTRF assay using 1 μM forskolin. Results are the means and SEM of three measurements performed in triplicate. Student’s *t*-test or one-way ANOVA determined statistical significance. ^ns^ *p* > 0.99, * *p* < 0.05, ** *p* < 0.01 and **** *p* < 0.0001.

HEK293 and ΔGRK2 cells stably expressing T2R14 were preincubated with or without beef peptides for 15 min. The cells were then stimulated with 0.5 mM DPH for 15 min, and the intracellular cAMP concentration was measured using the HTRF cAMP detection assay. Results are expressed as the means and SEM of three independent experiments, each performed thrice (Figure 4A,B). There was a significant difference in basal cAMP levels between the T2R14-WT and ΔGRK2 + T2R14 groups, upon 1 µM forskolin treatment. However, agonist treatments, both synthetic and beef peptides, showed no significant changes in cAMP production (Figure 4A). GRK2 also had no impact on this second messenger signaling. Conversely, antagonist beef peptide treatments did not significantly alter cAMP levels, regardless of the presence or absence of GRK2 (Figure 4B).

## 4. Discussion

GPCR desensitization begins when their cytosolic residues, including serine and threonine, are phosphorylated by specific kinases [11]. However, the mechanism and physiological importance of taste desensitization remain largely unclear. Our previous research showed that T2R14 triggers an innate immune response to certain microbial products, while T2R4 undergoes desensitization when exposed to bitter ligands such as quinine [42].

Agonist binding to Gi-coupled GPCRs activates the receptor, leading to inhibition of adenylyl cyclase, which decreases cAMP production, and often stimulates calcium signaling mediated by the βγ-subunit through phospholipase C activation. This study focused on the canonical T2R signaling pathway (T2R14-Gβγ-PLCβ-dependent intracellular calcium release) by measuring calcium release in cells after treatment with bitter agonists, antagonists and peptides, as well as on the role of GRK2 in delaying the T2R14 signal (desensitization) induced by beef peptides. Our results indicated that, out of eight beef peptides tested against T2R14, few displayed agonistic or antagonistic activity against DPH-induced responses (Figure 1 and Figure 2). Recent research has shown that activating HEK293T cells expressing T2R16 results in an increase in intracellular calcium when stimulated with bitter peptides from Jinhua ham [43]. Similarly, our earlier studies demonstrated calcium mobilization as a functional response of T2Rs (T2R4, T2R7, and T2R14) upon activation by beef and hen peptides, respectively [34,44]. However, pre-treatment with beef peptides did not significantly alter cAMP production (Figure 4). Conversely, changes in intracellular calcium signaling are evident. Therefore, we may speculate that calcium signaling is the predominant mechanism in the beef peptide-mediated T2R14 signaling pathway. T2R14 is a broadly tuned bitter receptor that binds a wide range of chemically diverse ligands; many bitter drugs, such as diphenhydramine, flufenamic acid, and apigenin, can activate it. Modeling studies suggest it has multiple orthosteric and allosteric binding modes. T2R14 interacts with various G proteins, including Gαgustducin-like and Gαi, and can regulate cAMP, Ca^2+^, NO, and other downstream signaling pathways. Consequently, in different tissues like airway smooth muscle, epithelium, and immune cells, the same agonist can produce different functional responses. If peptides are not highly selective, they may also activate other T2Rs or unrelated GPCRs, especially at higher concentrations. This can lead to unintended activation of bitter receptors in other tissues such as the gut and endocrine organs, potentially causing unpredictable immune or endocrine effects.

There are two opposing hypotheses regarding the mechanisms of desensitization of T2Rs. The first hypothesis proposes that bitter tastants like quinine interact with and inhibit GRKs, delaying T2R signal termination [13]. The second hypothesis proposes that GRKs might be involved in T2R phosphorylation and cause T2R signal termination [14]. Knocking down GRK impairs receptor phosphorylation, reducing β-arrestin recruitment and receptor desensitization. This typically results in prolonged and enhanced Gi signaling, causing more sustained inhibition of cAMP levels and potentially longer-lasting or increased calcium responses. Here, we investigated the ability of the beef-derived peptides to inhibit the physiologically important receptor, T2R14, and the role of GRK2 in T2R desensitization. We used CRISPR/Cas9 to delete the GRK2 gene to compare the effect of beef-derived peptides to synthetic T2R14 agonists and antagonists on the T2R14 desensitization (Figure 3). To understand the role of GRK2 in beef peptide-stimulated T2R14 signaling we measured the dynamics of change in intracellular Ca^2+^ (Figure 3). Further, we tested the pharmacological inhibition of GRK2 using cmpd101, which is selective for both GRK2/3. Cmpd101 treatment mimics the ΔGRK2 phenotype with respect to calcium mobilization of the area under the curve; however, the calcium signaling dynamics (rise and fall time) are different. This can be due to many reasons, including the off-target effects of Cmpd101. These results reinforce the advantage of using knockout models rather than relying on synthetic compounds to inhibit proteins of interest.

Our results suggested that deletion of the GRK2 can delay the T2R14 desensitization upon treatment with bitter peptides (Figure 3). The precise timing of Ca^2+^ signaling is a central aspect of many cellular processes, and Gαq and Gαi -coupled receptors are one of the primary drivers of intracellular Ca^2+^ release via activation of PLC-β and subsequent gating of inositol 1,4,5-trisphosphate (IP3) receptors [45]. These results demonstrate that the beef peptide-mediated delay in T2R14 desensitization depended on its ability to inhibit GRK2 intracellularly. Further research is needed to elucidate the mechanism (s) by which these peptides inhibit GRK2’s kinase activity and the involvement of other kinases like GRK3, GRK5 and GRK6 in influencing the Ca^2+^ signaling dynamics upon beef peptide stimulation. GRK2 is present in taste-bud cells, but the desensitization pathways of the taste T1Rs/T2Rs need to be clarified before further investigation of this phenomenon can proceed. The potential implications of these results on post-receptor signaling pathways, such as the MAPK pathway [46] and consequently additional downstream pathways, should also be explored. Furthermore, the β-arrestin recruitment modulated by the peptides needs to be explored in the future. Gaining insight into these mechanisms may help inform strategies to modify dietary behaviors, develop functional foods, and manipulate taste responses to improve health and nutrition.

## 5. Conclusions

The presence of T2Rs in many extraoral tissues suggests they may have important physiological roles beyond bitter taste perception [47]. In conclusion, this study demonstrates the potential of beef-derived peptides as antagonists of T2R14. Antagonistic peptides can be used to reduce unpleasantness and thereby improve the palatability of health-promoting bitter foods, such as fruit and vegetable extracts, as well as bitter medications. This work focused exclusively on GRK2 and examined the inhibitory effects of these food protein-derived peptides on T2R14 desensitization by measuring the kinetics of intracellular calcium signaling. A limitation of this study is that the possible involvement of other GRKs and kinases in food peptide-induced desensitization was not explored, which presents an avenue for future research. Additionally, real-time changes in cAMP response should be measured during T2R14 desensitization induced by food peptides.

## Data Availability

The original contributions presented in the study are included in the article/Supplementary Materials, further inquiries can be directed to the corresponding author.

## Supplementary Materials

The following supporting information can be downloaded at: https://www.mdpi.com/article/10.3390/nu18060901/s1, Figure S1: Analysis of cells surface expression of FLAG-bitter taste receptor 14 (293 + T2R14) and ΔGRK2 + T2R14; Figure S2: Effect of GRK2 inhibitor cmpd101 on T2R14 signal kinetics with agonistic and antagonistic beef peptides; Figure S3: Effect of GRK2 on signal kinetics with agonistic and antagonistic beef peptides.

## Abbreviations

The following abbreviations are used in this manuscript:

T2Rs	Bitter taste receptors
RFU	Relative fluorescence unit
DPH	Diphenhydramine
cAMP	Cyclic amino monophosphate
