# Supplementary material for: Beef-Derived Peptides Mediated Desensitization of Bitter Taste Receptor T2R14 Through GPCR Kinase 2
Source: Nutrients. 2026 Mar 12;18(6):901. doi: 10.3390/nu18060901 (PMC13029621; doi:10.3390/nu18060901)
Supplement: Supplementary file 1 [file nutrients-18-00901-s001.zip › nutrients-4157616-supplementary.pdf]

Supplementary Figures

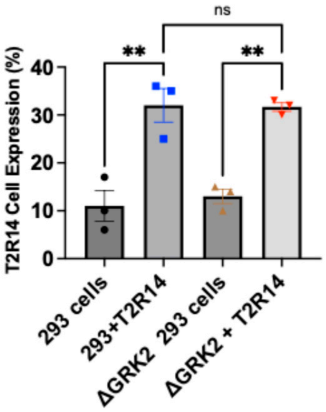

Figure S1. Analysis of cells surface expression of FLAG-bitter taste receptor 14 (293+T2R14) and ΔGRK2+T2R14. HEK293 cells stably expressing T2R14 in both T2R14-Wt and ΔGRK2+T2R14 were incubated with APC-conjugated FLAG antibody (1:500 dilution), and the cell surface expression are presented as percentage T2R14 cell surface expression. The data represent 3 independent experiments performed in duplicates. One-way ANOVA analysis was used, and statistical significance was calculated between the groups using Tukey's multiple comparison test (**$P < 0.01$).

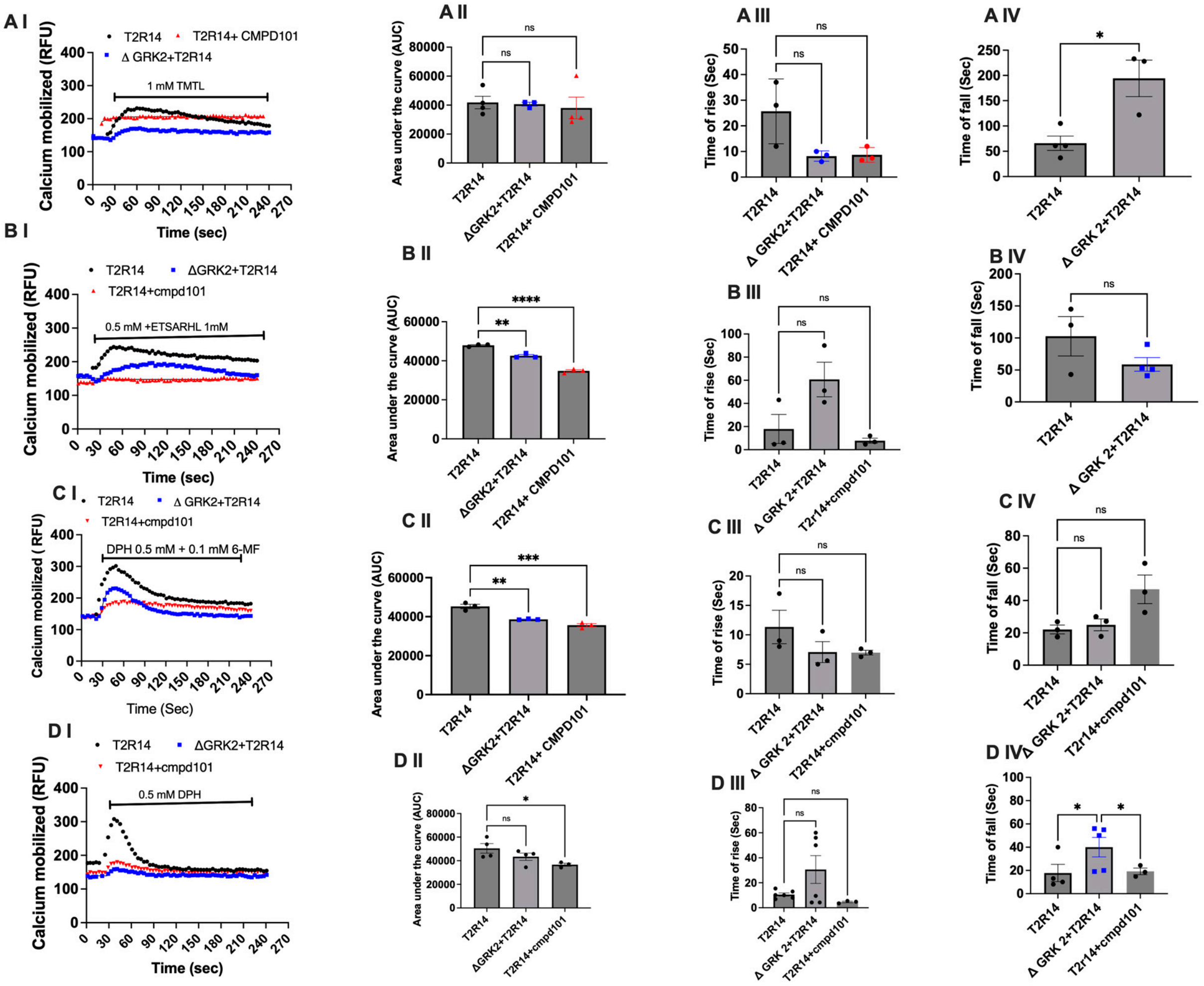

Figure S2. Effect of GRK2 inhibitor cmpd101 on T2R14 signal kinetics with agonistic and antagonistic beef peptides. HEK293 cells stably expressing T2R14 were treated with 0.01 mM cmpd101 for 15-20 mins at room temp. DPH (0.5 mM) and beef peptides (1 mM alone or in combination). Calcium mobilization assay was performed using the Fluo-4NW dye and RFU subtraction in HEK293 and Δ GRK2 cells expressing T2R14 or Δ GRK2 + T2R14, and the results were plotted as ΔRFU. The data represent the SEM of at least three independent experiments performed in triplicate.

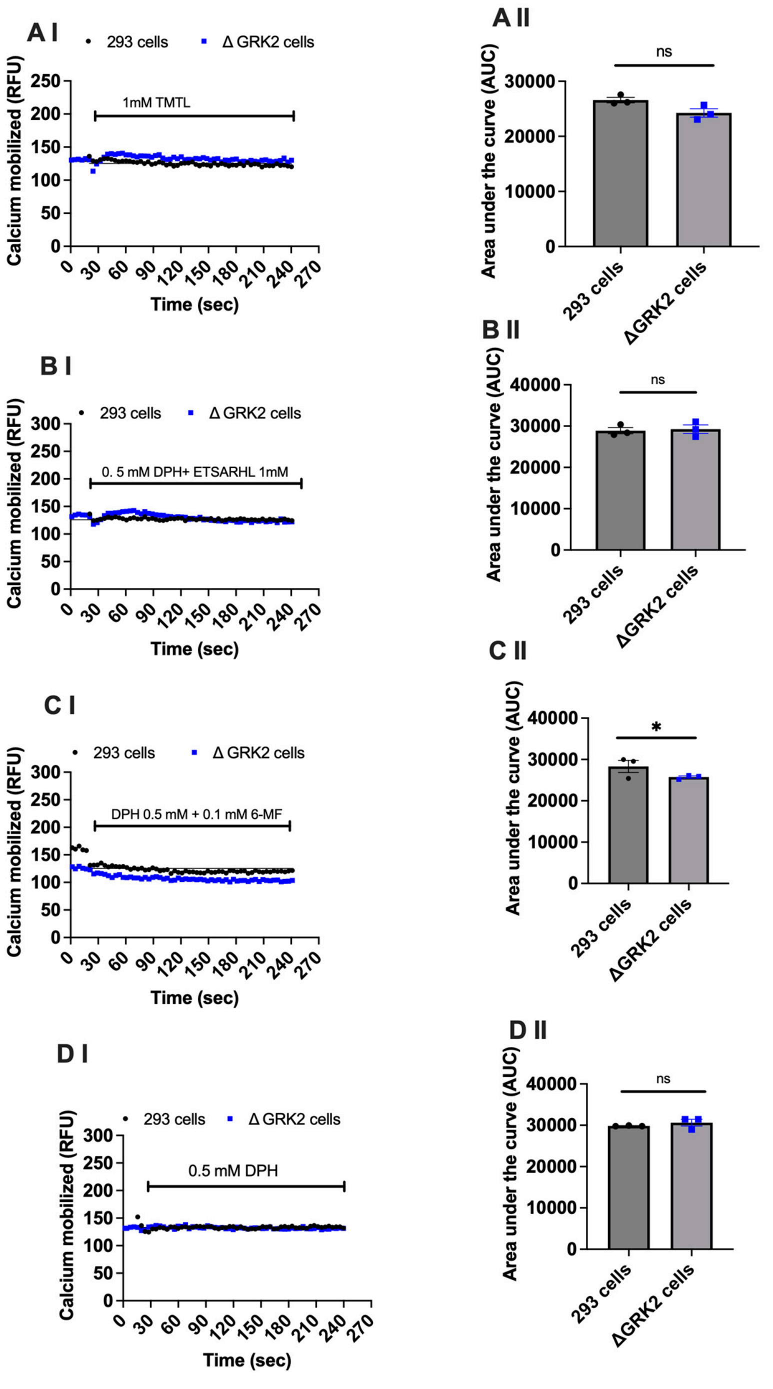

Figure S3. Effect of GRK2 on signal kinetics with agonistic and antagonistic beef peptides. HEK293 and ΔGRK2 cells were treated with DPH (0.5 mM) and beef peptides (1 mM alone or in combination). Calcium mobilization was assayed using the Fluo-4NW dye and continuously monitored over 300 sec using a Flex Station 3 multimode plate reader. Results are presented as relative fluorescence units (RFU) (AI-DI). **AII-DII**, area under the curve (AUC). The data represent the SEM of at least three independent experiments performed in triplicate.
